# Analysis of Ambulatory Trauma in a District General Hospital: System Delays and Need for an Evidence-Based Pathway

**DOI:** 10.7759/cureus.108924

**Published:** 2026-05-15

**Authors:** Joseph Latham, Faisal Mohammed, Oliver Hadfield, Oliver Lane, Benedict Sweetman, Rachel Dhir, Siwan Jones, Siva Santhanam, Satya Pydah

**Affiliations:** 1 Trauma and Orthopedics, The Grange University Hospital, Cwmbran, GBR; 2 Trauma and Orthopedics, Ysbyty Gwynedd, Bangor, GBR; 3 Surgery, University Hospital of Wales, Cardiff, GBR

**Keywords:** ambulatory, factors, pathway, trauma, waiting times

## Abstract

Background and aim

Ambulatory trauma represents a significant proportion of the orthopedic workload within the UK. Objective delays within ambulatory trauma pathways are poorly documented. This study aimed to quantify delays to surgery and identify modifiable factors to improve ambulatory trauma pathways within a district general hospital.

Methods

A retrospective analysis was conducted of adult ambulatory trauma patients requiring operative management without admission at a single DGH between September 2023 and January 2024 (n = 72). Overall delay (injury to surgery) and system delay (SD; booking to surgery) were analyzed. Subgroup analyses assessed injury type, subspecialist involvement, booking pathway, and theater setting.

Results

The mean SD was 4.5 days. Subspecialist involvement was associated with increased delay (4.8 vs 3.3 days, p = 0.02). Patients listed via the fracture clinic experienced longer delays than those booked directly from trauma meetings (7.3 vs 4.4 days, p = 0.18). Surgery on elective lists was associated with longer delays compared with trauma lists (6.2 vs 4.2 days, p = 0.03).

Conclusions

Ambulatory trauma patients experience significant delays to surgery. Delays were associated with the type of injury, need for subspecialty surgeons, and being listed on an elective list. These findings highlight current service provision deficiencies and the need for streamlined ambulatory trauma care pathways based on objective modifiable factors.

## Introduction

Ambulatory trauma accounts for approximately one-third of all trauma presentations in the UK [1]. It most commonly affects individuals aged 18-59 years [1], representing the primary working population, with an average of 1.09 dependents per earner in the UK (Office for National Statistics) [2]. Despite this significant socioeconomic impact, there is limited literature examining ambulatory trauma care within district general hospitals (DGHs) throughout the UK.

Ambulatory trauma pathways are central to maintaining patient flow within DGHs, aiming to reduce inpatient admissions while facilitating timely assessment and management [3]. However, these pathways are frequently subject to delays at multiple stages, including triage, imaging, and definitive treatment. Patients are often assigned lower clinical priority, which may result in prolonged waiting times and fragmented care delivery [1,3].

Such inefficiencies can adversely affect patient outcomes, patient experience, and overall service performance. In the context of rising demand and ongoing orthopedic backlogs, delays within ambulatory pathways place additional strain on already stretched healthcare resources [4].

This study aims to evaluate current ambulatory trauma processes within a DGH by quantifying overall and system delays (SDs) to operative intervention, identifying key modifiable factors contributing to these delays, and highlighting the need for a standardized pathway to improve efficiency and patient flow, with potential applicability across the NHS. Specifically, the primary objective was to quantify overall and SDs to surgery in ambulatory trauma, while the secondary objective was to assess whether subspecialist involvement, booking route, and theater list were associated with delay.

## Materials and methods

For the purposes of this study, ambulatory trauma was defined as acute orthopedic injury requiring operative management without overnight admission, managed on a same-day or planned day-case basis. All adult ambulatory trauma patients attending Ysbyty Gwynedd between September 16, 2023, and January 7, 2024, who required operative management without inpatient admission were included. Patients were identified consecutively from trauma meeting records throughout the study period.

Patients under 18 years of age and those with open fractures were excluded. Fracture-clinic bookings were cross-checked against theater booking records to ensure capture of clinic-listed ambulatory cases. Seventy-two patients met the inclusion criteria.

Data collected included date of injury, anatomical site of injury, date of surgery, date of booking, mode of booking, subspecialist involvement, and location of surgery (elective vs trauma theaters). The overall delay (OD) was defined as the days from the date of injury to the surgery date, while the SD was from the booking date to the surgery date.

At our hospital, ambulatory trauma patients are typically splinted, provided with analgesia and fasting instructions, and discussed with the on-call consultant. The unit operates a single daily trauma theater, which also accommodates neck of femur fracture operations. Currently, ambulatory trauma procedures are scheduled based on theater availability and surgeon subspecialty availability.

Data collection

All data were collected from trauma meetings and booking departments, anonymized, and recorded internally using Microsoft Excel (Microsoft Corporation, Redmond, WA, USA).

Statistical analysis

Statistical analysis was performed using Microsoft Excel (Microsoft Corporation), applying Student’s t-test, with statistical significance defined as p < 0.05.

Ethical approval

This retrospective service evaluation used anonymized, routinely collected data and did not require formal ethical approval.

## Results

A total of 72 ambulatory trauma patients requiring operative management without inpatient admission were included in the analysis. Across all cases, the mean OD was 8.2 days, while the SD was 4.5 days.

Delays by injury type

Delays were further subgrouped by injury type. Hand and wrist trauma represented the largest proportion of cases (30, 41.7%), followed by foot and ankle injuries (15, 20.8%). Elbow and knee injuries each accounted for 11 cases (15.3%), while shoulder trauma was the least common (5, 6.9%). The longest OD was observed in knee injuries (mean 11.1 days), followed by non-distal radius fractures (9.0 days), elbow injuries (8.6 days), and foot and ankle injuries (8.5 days). Shorter delays were seen in distal radius fractures (5.7 days) and shoulder injuries (4.0 days). In terms of SDs, the greatest delays were seen in foot and ankle and elbow injuries (both 6.3 days), followed by knee injuries (4.5 days) and distal radius fractures (4.4 days). Shoulder injuries had shorter delays (3.4 days), with the shortest delays observed in non-distal radius fractures (2.2 days) (Table 1).

**Table 1:** Delays to surgery grouped by injury type OD, overall delay; SD, system delay

Injury type	Number of cases	OD (mean days)	SD (mean days)
Hand and wrist	30	7.4	3.3
Distal radius	15	5.7	4.4
Non-distal radius	15	9	2.2
Foot and ankle	15	8.5	6.3
Elbow	11	8.6	6.3
Knee	11	11.1	4.5
Shoulder	5	4	3.4

Delays due to subspecialist involvement

Consultant subspecialty involvement was documented in 55/72 cases. Of these, 32/55 cases (58.2%) were performed by a subspecialist surgeon. Cases requiring subspecialist involvement had a mean SD to surgery of 4.8 days, compared with 3.3 days in cases not requiring subspecialist input. This difference was statistically significant (p = 0.02), suggesting an association between subspecialist involvement and increased delay to theater (Table 2).

**Table 2:** Comparison of delay to theater when the operating consultant is a specialist or a nonspecialist SD, system delay

Group	Total cases	Mean SD (days)
Specialist	32	4.8
Nonspecialist	23	3.3
t-Test p-value	0.02	-

Delays to the booking pathway

Most patients (61/72, 84.7%) were booked directly for surgery from the emergency trauma team. These patients experienced a mean delay to surgery of 4.4 days. In contrast, patients booked from the fracture clinic (11/72, 15.3%) experienced a longer mean delay of 7.3 days (p = 0.18), although this difference did not reach statistical significance (Table 3).

**Table 3:** Comparison of delay to theater when booked from trauma meeting or from clinic SD, system delay

Group	Total cases	Mean SD (days)
Booked from the trauma meeting	61	4.4
Booked from the clinic	11	7.3
t-Test p-value	0.18	-

Delays due to theater location (elective vs trauma lists)

Patients operated on a dedicated trauma list experienced a mean delay to surgery of 4.2 days, compared with 6.2 days for patients operated on an elective list. This difference was statistically significant (p = 0.03) (Table 4).

**Table 4:** Comparison of delay to theater when surgery is carried out on a trauma or an elective list SD, system delay

Group	Total cases	Mean SD (days)
Trauma list	57	4.2
Elective list	15	6.2
t-Test p-value	0.03	-

## Discussion

This study reinforces that adult ambulatory trauma patients experience significant delays in treatment. The OD was 8.2 days, while the SD was 4.5 days, reflecting a substantial interval between injury, decision-making, and operative intervention. The OD from date of injury to date of surgery is the most important metric when considering overall patient experience [5-8], whereas the SD from booking date to surgery date reflects inefficiencies in service provision [9]. These figures highlight the cumulative impact of service inefficiencies on the overall experience and care of ambulatory patients. We analyzed these inefficiencies in terms of specific criteria: type of injury and subspecialty involvement, booking pathway, and operation setting (trauma vs elective theater).

Type of injury

Knee injuries experienced the longest OD, with a mean time of 11.1 days, exceeding all other anatomical sites. This was followed by elbow injuries (8.6 days) and foot and ankle injuries (8.5 days), suggesting lower prioritization, pathway inefficiencies, or resource constraints in these subgroups. In contrast, shoulder injuries and hand and wrist injuries had the shortest OD (4.0 and 7.4 days, respectively), suggesting higher prioritization or more streamlined pathways for these upper limb cases (Table 1). The longest SD was seen in foot and ankle and elbow injuries, both with a mean delay of 6.3 days. Knee injuries also demonstrated a notable SD of 4.5 days, while hand and wrist injuries had the shortest SD of 3.3 days. This highlights inherent variability in operative access depending on the anatomical site of injury, which is influenced by the British Orthopaedic Association’s standards for operative timing [10].

Subspeciality involvement

Subspecialty involvement was identified as a significant factor associated with increased SD. Patients requiring subspecialty input experienced a longer mean delay compared with those managed by nonspecialist consultants (4.8 vs 3.3 days, p = 0.02) (Table 2). This finding highlights the impact of subspecialty availability on timely access to surgery within a DGH.

Workforce limitations and subspecialization represent important challenges that are often multifactorial and not easily addressed. While subspecialty expertise is essential for selected cases, reliance on limited specialist availability may be associated with delays in treatment, with potential consequences for patient experience and quality of care.

These findings suggest that early identification and allocation of subspecialty input are important but should be balanced against the risk of treatment delay. When designing ambulatory trauma pathways, careful consideration should therefore be given to whether subspecialty involvement is essential to optimize efficiency without compromising clinical outcomes.

Booking pathways

Another important factor identified was booking pathways. Our study demonstrated that patients listed directly from the trauma multidisciplinary team (MDT) experienced shorter delays to surgery compared with those referred to the fracture clinic prior to booking (4.4 vs 7.3 days, p = 0.18) (Table 3). Although this difference did not reach statistical significance, the observed trend suggests a potential benefit of direct listing from the trauma MDT, which may facilitate earlier access to surgery and improve patient experience.

In addition, avoiding unnecessary fracture clinic review may help reduce outpatient workload and optimize resource utilization. Although this relationship is intuitive, the absence of formalized ambulatory trauma pathways means that some patients potentially continue to undergo clinic review prior to operative decision-making, contributing to avoidable delays. This represents a possible modifiable factor in improving pathway efficiency.

Theater setting (trauma vs elective list)

In terms of theater setting, patients scheduled on trauma lists experienced shorter delays to surgery compared with those on elective lists (4.2 vs 6.2 days, p = 0.03) (Table 4). This is not unexpected, as trauma lists are structured to prioritize urgent and semi-urgent cases, allowing more timely access to surgery.

The availability of elective theater capacity remains valuable in managing ambulatory trauma workload; however, this appears to be associated with increased delays, likely reflecting differences in scheduling and prioritization. Cases allocated to elective lists are often those deemed less urgent or requiring subspecialty input and may therefore be associated with longer waiting times.

These findings highlight the importance of balancing the use of both trauma and elective theater capacity. While elective lists provide an additional option, consideration should be given to the impact on waiting times, with appropriate prioritization required to minimize avoidable delay.

Clinical implications

Our findings have identified key modifiable factors associated with delay in ambulatory trauma care. Modifiable factors include subspecialty involvement and theater location, whereas the anatomical injury site is non-modifiable. We have demonstrated that ambulatory care may be streamlined by involving a specialist only when essential and using trauma lists when possible, while acknowledging that elective lists are suitable for certain less urgent cases. These findings provide objective parameters to consider when designing an effective ambulatory trauma pathway for a DGH.

Limitations

This study is limited by its retrospective design, modest sample size, and reliance on recorded documentation. Additionally, consultant subspecialty data were not available for all cases, and delays may have been influenced by unmeasured patient or system confounding variables.

The study period, spanning September 2023 to January 2024, encompasses autumn and winter months only. Seasonal variation in trauma volume, staffing levels, and theater availability may therefore influence the findings, and results may not be representative of patterns seen during spring and summer months. Replication across a full calendar year and across multiple centers would strengthen the generalizability of these findings.

A significant contributor to the delay in ambulatory trauma management is the inclusion of neck of femur fractures on shared trauma operating lists. Although nonambulatory, these cases require surgery within 48 hours in accordance with NICE guidelines and are therefore prioritized [11]. This may reduce theater availability for ambulatory trauma, contributing to delays within the pathway. This represents a potential confounding factor affecting pathway efficiency that is not directly captured within our dataset.

Although data distribution was assessed visually prior to analysis, some variables may not have been normally distributed, which represents a limitation of the statistical analysis.

## Conclusions

Ambulatory trauma patients experience significant delays to surgery. Delays were associated with the type of injury, need for subspecialty surgeons, and being listed on an elective list. These findings highlight current service provision deficiencies and the need for streamlined ambulatory trauma care pathways based on objective findings.
